# Association Between Coffee Consumption and Glucose Metabolism Markers in Korean Adults

**DOI:** 10.3390/nu17091484

**Published:** 2025-04-28

**Authors:** Sooyeun Choi, Taeyoung Park, Youjin Je

**Affiliations:** 1 Department of Food and Nutrition, Kyung Hee University, 26 Kyunghee-Daero, Dongdaemun-Gu, Seoul 02447, Republic of Korea; isubject0519@naver.com; 2 Department of Applied Statistics, Yonsei University, 50 Yonsei-Ro, Seodaemun-Gu, Seoul 03722, Republic of Korea

**Keywords:** coffee, black coffee, coffee with sugar and/or cream, glucose metabolism markers, insulin resistance

## Abstract

**Background/Objectives:** Studies examining the association between coffee consumption and glucose metabolism markers have reported inconsistent findings, and few have considered coffee type. We investigated the association between coffee consumption and glucose metabolism markers in Korean adults. **Methods:** A cross-sectional analysis was conducted using data from the Korea National Health and Nutrition Examination Survey (2019–2021), including 7453 adults aged 19–64 years. Coffee consumption was assessed using a 24 h dietary recall and categorized as black coffee or coffee with sugar and/or cream (non-drinkers, ≤1 cup/day, 2 cups/day, and ≥3 cups/day). Multivariable logistic regression models were used to assess the associations with glucose metabolism markers, including the homeostasis model assessment of insulin resistance (HOMA-IR). **Results:** The mean age of participants was 40.6 years (standard error: 0.19). After full adjustment for covariates, in women, consuming two or more cups of black coffee per day was inversely associated with elevated HOMA-IR (2 cups/day: OR = 0.73, 95% CI: 0.56–0.96; ≥3 cups/day: OR = 0.66, 95% CI: 0.44–0.99) and fasting insulin levels (2 cups/day: OR = 0.70, 95% CI: 0.54–0.91; ≥3 cups/day: OR = 0.64, 95% CI: 0.43–0.93), compared to no consumption, showing a significant linear trend (*p*-trend ≤0.01 for all cases). By coffee type, women who consumed more black coffee had lower odds of elevated HOMA-IR and fasting insulin levels (*p*-trend ≤0.02). In men or those consuming coffee with sugar and/or cream, no significant associations with glucose metabolism markers were observed. **Conclusions:** Our findings indicate that consuming two or more cups of black coffee per day is inversely associated with insulin resistance in Korean women.

## 1. Introduction

With a wide range of customization options, coffee remains one of the most widely consumed beverages worldwide and may significantly impact human health at the population level. In 2019, Koreans consumed an average of 315 g of total beverages per day, with coffee being the largest component at 108 g [1]. A meta-analysis of 30 prospective studies suggested that the risk of type 2 diabetes mellitus (T2DM) decreases by 6% per additional cup of coffee consumed per day, with similar findings for both caffeinated and decaffeinated coffee [2]. However, the physiological pathways underlying the inverse association between coffee consumption and T2DM risk remain unclear. Insulin resistance is a major contributor to T2DM pathogenesis and plays a critical role in metabolic dysfunctions, such as metabolic syndrome and dyslipidemia [3]. The homeostasis model assessment of insulin resistance (HOMA-IR) is a reliable method for assessing insulin resistance in large-scale epidemiological studies [4,5]. A recent meta-analysis reported that elevated HOMA values are associated with an increased risk of metabolic diseases, including T2DM and systemic arterial hypertension [6]. The Mediterranean diet, characterized by a high intake of vegetables, extra-virgin olive oil, and polyphenol-rich foods, has been associated with greater improvements in insulin resistance in individuals with obesity compared to other dietary patterns [7]. Coffee, a major source of polyphenols [8], has demonstrated improvements in insulin sensitivity in vitro and in vivo [9,10]. It contains diverse bioactive compounds, including caffeine; phenolic compounds such as chlorogenic acid and caffeic acid; and diterpenes such as cafestol and kahweol, all of which exhibit anti-inflammatory and antioxidant properties in vitro and in vivo [11,12,13]. Taken together, coffee consumption appears to be associated with glucose metabolism markers, including insulin resistance.

However, studies examining the relationship between coffee consumption and glucose metabolism markers, including HOMA-IR, have reported inconsistent findings [14,15,16,17,18,19,20,21,22,23,24,25]. A meta-analysis of seven randomized controlled trials (RCTs) indicated that acute caffeine ingestion reduces insulin sensitivity in healthy individuals [26]. Additionally, categorizing coffee types allows the investigation of the specific effects of coffee constituents on health outcomes. Instant coffee mix, which constitutes a substantial portion of the Korean coffee market, typically contains significant levels of sugar and saturated fatty acids [27]. Consumption of instant coffee mixes has been positively associated with metabolic syndrome [27]. However, few studies on the relationship between coffee consumption and glucose metabolism markers have considered coffee type, which may contribute to inconsistent findings. Furthermore, no Korean studies have examined the associations between coffee intake and glucose metabolism markers, including HOMA-IR, while considering coffee type.

Therefore, we aimed to investigate the associations between coffee consumption and glucose metabolism markers (fasting glucose, fasting insulin, HbA1c levels, HOMA-IR, and HOMA-β) in Korean adults, considering coffee type, using data from the Korea National Health and Nutrition Examination Survey (KNHANES).

## 2. Materials and Methods

### 2.1. Study Population

We conducted the present study using data from the eighth period (2019–2021) of the KNHANES, a nationally representative, cross-sectional survey conducted annually by the Korea Disease Control and Prevention Agency (KDCA) under the Ministry of Health and Welfare in the Republic of Korea. The KNHANES includes non-institutionalized Korean citizens and employs a multi-stage clustered probability sampling design. Data were collected from 22,559 participants in the KNHANES during 2019–2021. Among them, we included 17,881 participants who completed all three components of the KNHANES (health interview, health examination, and nutrition survey). We then excluded the following participants, in order: 7595 participants who were <19 or ≥65 years old; 1239 participants who self-reported a diagnosis of stroke, myocardial infarction/angina, renal diseases, cancer, or diabetes mellitus, or who were taking medications for diabetes mellitus; 42 participants who were pregnant; 27 participants who were lactating; 285 participants who had fasted for less than 8 h at the time of blood testing; 145 participants who had extreme total daily energy intake (<500 or >5000 kcal/day); 2 participants with missing data on fasting plasma glucose, fasting insulin, or HbA1c levels; 814 participants with missing data on smoking status, alcohol consumption, or physical activity; and 279 participants who were following diet therapy for disease. As a result, we included 7453 participants, comprising 4155 women and 3298 men.

**Figure 1:**
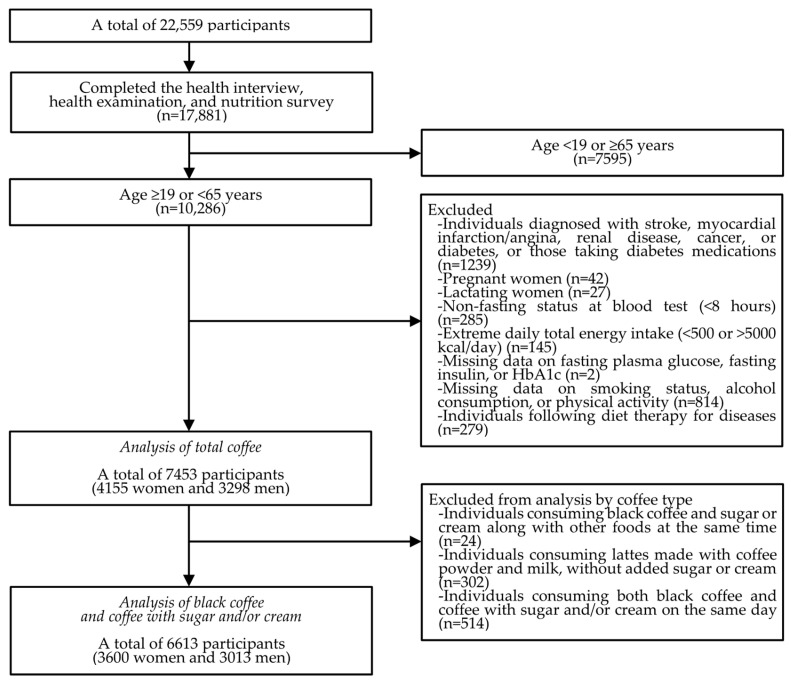
Study population aged 19–64 years after applying the exclusion criteria.

For the analysis by coffee type, we additionally excluded the following participants, in order: 24 participants who consumed black coffee and sugar or cream along with other foods at the same time, as we were not able to determine whether the sugar or cream was added to the black coffee or to the other foods; 302 participants who consumed lattes made with coffee powder and milk without added sugar or cream, as the number of such participants was too small to be included in the coffee type analysis; and 514 participants who consumed both black coffee and coffee with sugar and/or cream on the same day. Consequently, 6613 participants, comprising 3600 women and 3013 men, were included in the analysis by coffee type. A flow diagram illustrating the inclusion process for the study population is presented in Figure 1. The Institutional Review Board of the KDCA approved the KNHANES datasets, and all participants provided informed consent to the KDCA (2018-01-03-C-A [approval date: 19 December 2018], 2018-01-03-2C-A [approval date: 26 June 2020], 2018-01-03-5C-A [approval date: 23 April 2021]).

### 2.2. Assessment of Coffee Consumption

We evaluated coffee consumption using 24 h dietary recall data from KNHANES. Coffee types were categorized as black coffee or coffee with sugar and/or cream. To address potential errors in summing food intake across varying physical states, we used food intake data for the tertiary food code provided in the KNHANES dataset, calculated using solid-content-based conversion coefficients. In KNHANES, coffee powder is designated as the reference food for the tertiary food code of coffee. The weight of one sachet of instant black coffee powder (2.1 g) was defined as the dry weight equivalent of one cup of coffee (180 mL), based on a commercial ratio. The food intake data for the tertiary food code of coffee with sugar and/or cream include the weights of sugar and/or cream along with that of the coffee powder. In our analyses, we considered only the weight of the coffee powder, which was calculated based on the coffee powder content (%) determined from the commercial product names listed in the dataset. If the coffee product name was not provided or if the coffee powder content (%) could not be determined, we used the median coffee powder content (%) of the remaining coffee products. Based on previous research [28] and the distribution of coffee intake in our study population, we categorized coffee consumption into the following groups: non-drinkers, ≤1 cup/day (0<–2.1 g), 2 cups/day (2.1<–4.2 g), and ≥3 cups/day (>4.2 g).

### 2.3. Assessment of Glucose Metabolism Markers

Plasma glucose levels were enzymatically measured using hexokinase with the Labospect 008AS (Hitachi, Tokyo, Japan) and Qualigent GLU (Sekisui, Tokyo, Japan) at the Seegene Medical Foundation in Seoul, Republic of Korea. Insulin levels were measured using electrochemiluminescence immunoassay with the Modular E801 (Roche, Mannheim, Germany) and Elecsys Insulin (Roche, Mannheim, Germany). HbA1c levels were measured using high-performance liquid chromatography with the Tosoh G8 (Tosoh, Tokyo, Japan) and HLC-723G8 HbA1c-specific reagents (Tosoh, Tokyo, Japan). HOMA-IR was calculated using the formula (fasting plasma insulin [μIU/mL] × fasting plasma glucose [mmol/L])/22.5. Homeostatic model assessment of beta-cell function (HOMA-β) was calculated using the formula (20 × fasting plasma insulin [μIU/mL])/(fasting plasma glucose [mmol/L] − 3.5) [29]. Glucose metabolism markers were defined as follows, based on the National Cholesterol Education Program Adult Treatment Panel III, diabetes mellitus guidelines, and previous research [14,15,16,30,31,32]: hyperglycemia (fasting plasma glucose ≥ 5.55 mmol/L [≥100 mg/dL]); high fasting insulin (fasting insulin > 10.5 μIU/mL [75th percentile]); high HOMA-IR (HOMA-IR > 2.5 [75th percentile]); low HOMA-β (HOMA-β < 58.2 [25th percentile]); and high HbA1c (HbA1c ≥ 5.7%). The HOMA-IR cutoff of 2.5 aligns with the cutoff for metabolic syndrome proposed in previous research on the general Korean population [32]. In the KNHANES 2019–2021 data, insulin levels below 0.4 μIU/mL were recorded as “<0.4” in the categorical variable HE_insulin_etc, rather than as a value in the continuous variable HE_insulin. We converted ‘<0.4’ in the categorical variable to a numerical value of 0.39 μIU/mL in the continuous variable.

### 2.4. Covariates

Various demographic and lifestyle factors were evaluated through personal interviews or self-reported questionnaires. Education levels were categorized as middle school or lower, high school, and college or higher. Monthly household income was classified into quartiles. Marital status was categorized as married or unmarried. Alcohol consumption was calculated by multiplying the reported frequency of alcohol consumption in the past year by the amount of alcohol consumed per serving and was then categorized as <1, 1–<3, and ≥3 servings per day. Smoking status was classified as non-smoker, past smoker, and current smoker. Sleep duration was categorized into <6, 6–<8, and ≥8 h per day. Physical activity was categorized as low or high, with high physical activity defined as at least 150 min of moderate activity per week, at least 75 min of vigorous activity per week, or at least 150 min of a combination of moderate and vigorous activity per week, with 1 min of vigorous activity considered equivalent to 2 min of moderate activity. Arterial hypertension diagnosis was classified as ‘yes’ or ‘no’. Family history of diabetes mellitus was categorized as ‘yes’ if at least one parent or sibling had been diagnosed with diabetes mellitus and ‘no’ otherwise. Supplement intake was classified as ‘yes’ if dietary supplements were consumed on the day before the survey and ‘no’ otherwise. Diet quality was assessed using a modified diet quality index for Koreans (DQI-K) and categorized as good or poor. DQI-K points of ‘0 and 1’ or ‘0, 1, and 2’ were assigned to each of the eight dietary factors according to references such as the Korean Dietary Reference Intakes. The total DQI-K score, calculated as the sum of all factor points, ranged from 0 to 9, with scores of 0–4 indicating good diet quality and scores of 5–9 indicating poor diet quality [33]. Daily total energy intake (kcal/day) was measured using 24 h dietary recall data.

### 2.5. Statistical Analysis

For lifestyle and demographic variables, we obtained the prevalence (number and weighted percentage) for categorical variables. We calculated means with standard errors (SE) for normally distributed continuous variables and medians with interquartile ranges for non-normally distributed continuous variables, based on histograms, Q–Q plots, skewness, and kurtosis, given the large sample size of this study [34,35]. We compared participants’ characteristics across coffee consumption categories using the chi-squared test for categorical variables and analysis of variance (F-test) for continuous variables, with PROC SURVEYFREQ and PROC SURVEYREG, accounting for the multi-stage clustered probability sampling design of the KNHANES. To estimate the multivariable-adjusted odds ratios (ORs) and 95% confidence intervals (CIs) for glucose metabolism markers by coffee consumption, we used multivariable logistic regression models with the PROC SURVEYLOGISTIC procedure. Model 1 was adjusted for age (continuous) and sex (women and men). Model 2 included additional adjustments for the following variables: BMI (kg/m^2^, continuous), education level (middle school or lower, high school, and college or higher), monthly household income (lowest, lower-middle, upper-middle, and highest quartile), marital status (married or unmarried), alcohol consumption (<1, 1–<3, and ≥3 servings/day), smoking status (non-smoker, past smoker, and current smoker), sleep duration (<6, 6–<8, and ≥8 h/day), physical activity (low or high), arterial hypertension diagnosis (yes or no), family history of diabetes mellitus (yes or no), supplement intake (yes or no), DQI-K (poor diet quality [scores of 5–9] or good diet quality [scores of 0–4]), and total daily energy intake (kcal/day, continuous). When selecting covariates for inclusion in the models, we considered previous research related to this topic [14,16,17,18,20,36] and the characteristics of our study population.

We tested for potential effect modification by sex by adding cross-product terms to the fully adjusted models, as several previous studies have suggested sex-specific effects of coffee on insulin resistance [16,17]. Since we found significant interactions between coffee consumption and sex in the elevated HOMA-IR and fasting insulin analyses, we conducted stratified analyses by sex for these markers (*p*-interaction ≤ 0.04 for all cases). When we additionally tested for potential effect modification by age group (10-year intervals or dichotomized at age 45) or obesity (defined as BMI ≥ 25 kg/m^2^ [37]), we found no significant interaction between coffee intake and age group or obesity in any glucose metabolism marker analyses. Moreover, a prospective study indicated that HOMA-IR was more strongly associated with an increased risk of T2DM than other markers, including HOMA-β, fasting glucose, and insulin levels, after excluding individuals with fasting glucose ≥ 6.99 mmol/L (≥126 mg/dL) at baseline [38]. Accordingly, we conducted sensitivity analyses for HOMA-IR, restricting the study population to individuals with fasting glucose levels < 6.99 mmol/L (<126 mg/dL).

We conducted an additional analysis on the associations between coffee consumption and glucose metabolism markers among Korean adults aged 65 years and older. As the geriatric population often presents with frailty or multimorbidity, the impact of coffee intake may be masked, allowing other risk factors to play a more prominent role in late life glycemic parameters. Furthermore, despite the strict exclusion criteria in this study, older adults, who are more likely to be in preclinical stages of chronic diseases, may have reduced coffee intake, resulting in a higher potential for reverse causality. We found no significant interaction between coffee intake and sex among Korean older adults in any glucose metabolism marker analyses. The sample size of the older adults was insufficient to conduct a subgroup analysis by coffee type. The cutoffs for high HOMA-IR (HOMA-IR > 2.7 [75th percentile]), high fasting insulin (fasting insulin >10.7 μIU/mL [75th percentile]), and low HOMA-β (HOMA-β < 50.6 [25th percentile]) were recalculated within the study population of older adults for the subsequent logistic regression analyses. All other analytical procedures were identical to those used in the main analysis. Additionally, as shown in Figure 1, individuals under 19 years of age were excluded from this study due to potentially differing glucose metabolism compared to adults, including insulin resistance induced by significant changes in growth hormone/insulin-like growth factor I and sex hormone levels during puberty [39], and because about 95% of them were non-drinkers. All statistical analyses were performed using SAS software (version 9.4; SAS Institute Inc., Cary, NC, USA). A two-tailed *p*-value of less than 0.05 was considered statistically significant.

## 3. Results

### 3.1. General Characteristics

Table 1 presents the general characteristics of Korean adults according to total coffee consumption categories. The mean age of all participants was 40.6 years (standard error: 0.19). Compared with individuals in the lowest group (non-drinkers), those in the highest group of coffee consumption (≥3 cups/day) were more likely to be older, male, married, and have higher monthly household incomes. Those in the highest category of coffee consumption were also more likely to be heavy alcohol drinkers, have been diagnosed with arterial hypertension, consume supplements, and have poorer diet quality compared to those in the lowest category. Additionally, adults with higher coffee consumption had a higher BMI, higher education levels, greater daily total energy intake, less physical activity, and shorter sleep durations. Frequent coffee drinkers also tended to be current smokers.

### 3.2. Coffee Consumption and Glucose Metabolism Markers

Table 2 presents the results of multivariable logistic regression analyses on the associations between coffee consumption and glucose metabolism markers in Korean adults. After full adjustment for covariates, consuming 2 cups/day of total coffee showed suggestive inverse associations with high HOMA-IR and fasting insulin levels compared to no consumption (HOMA-IR: OR = 0.87, 95% CI: 0.73–1.05; fasting insulin: OR = 0.88, 95% CI: 0.73–1.06). By coffee type, consuming ≤1 cup/day of black coffee was inversely associated with high HOMA-IR (OR = 0.74, 95% CI: 0.58–0.95) and fasting insulin levels (OR = 0.79, 95% CI: 0.62–0.999), while consuming 2 cups/day of black coffee showed suggestive inverse associations (HOMA-IR: OR = 0.81, 95% CI: 0.64–1.03; fasting insulin: OR = 0.82, 95% CI: 0.64–1.04). Coffee with sugar and/or cream was not significantly associated with glucose metabolism markers.

**Figure 2:**
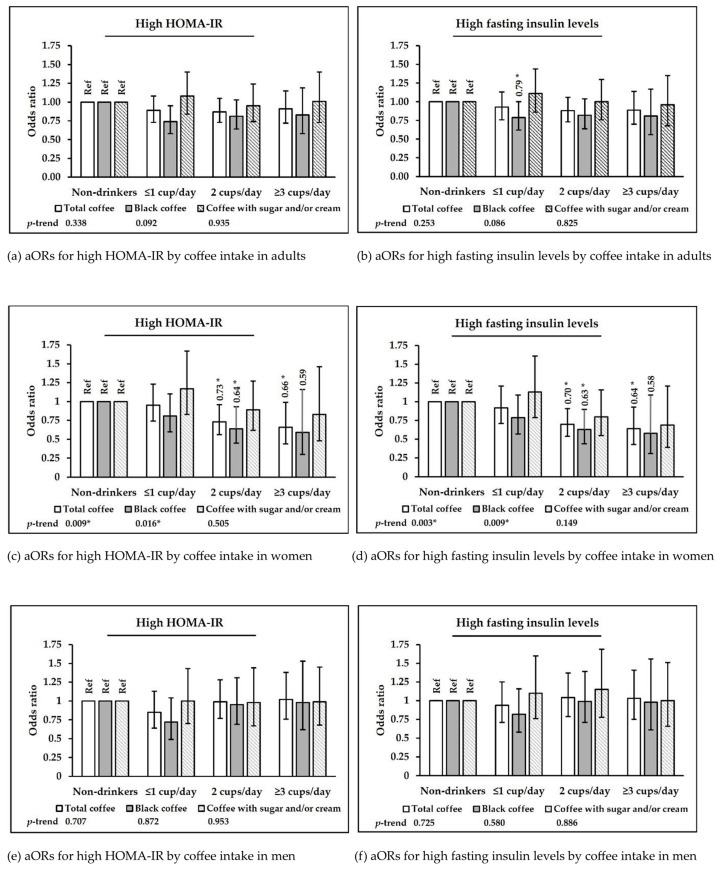
Multivariable-adjusted ORs and 95% CIs for high fasting insulin and HOMA-IR according to coffee consumption and type in Korean adults, women, and men aged 19–64 years. The asterisk (*) indicates statistical significance at *p* < 0.05.

Table 3 and Figure 2 show the associations between coffee consumption and high HOMA-IR and fasting insulin levels in Korean adults by sex. After full adjustment for covariates, in women, consuming ≥ 2 cups/day of total coffee was inversely associated with elevated HOMA-IR (2 cups/day: OR = 0.73, 95% CI: 0.56–0.96; ≥3 cups/day: OR = 0.66, 95% CI: 0.44–0.99; *p*-trend = 0.009) and fasting insulin levels (2 cups/day: OR = 0.70, 95% CI: 0.54–0.91; ≥3 cups/day: OR = 0.64, 95% CI: 0.43–0.93; *p*-trend = 0.003) compared to no consumption. By coffee type, women who consumed more black coffee had lower odds of elevated HOMA-IR (*p*-trend = 0.016) and fasting insulin levels (*p*-trend = 0.009), showing a significant linear trend. Consuming 2 cups/day of black coffee was inversely associated with elevated HOMA-IR (OR = 0.64, 95% CI: 0.45–0.93) and fasting insulin levels (OR = 0.63, 95% CI: 0.44–0.90). Consumption of ≥3 cups/day of black coffee showed suggestive inverse associations with elevated HOMA-IR (OR = 0.59, 95% CI: 0.30–1.16) and fasting insulin levels (OR = 0.58, 95% CI: 0.31–1.09), possibly due to the limited sample size in the highest intake group. Table S1 shows the associations between coffee consumption and high HOMA-IR and fasting insulin levels in Korean women, based on five sequential adjustment models. In men or those consuming coffee with sugar and/or cream, coffee consumption was not significantly associated with glucose metabolism markers.

Table S2 presents the results of sensitivity analyses on the associations between coffee consumption and elevated HOMA-IR in Korean adults, restricting the study population to individuals with fasting glucose levels < 6.99 mmol/L (<126 mg/dL). After full adjustment for covariates, the associations were consistent with the findings from the main analyses. Table S3 shows the results of additional analyses on the associations between coffee consumption and glucose metabolism markers in Korean adults aged 65 years and older. After full adjustment for covariates, older adults who consumed more coffee had lower odds of high HOMA-IR (*p*-trend = 0.023) and fasting insulin levels (*p*-trend = 0.009), showing a significant linear trend. However, this finding should be interpreted with caution, considering the general characteristics of geriatric populations (see Section 2.5).

## 4. Discussion

This study investigated the associations between coffee consumption and glucose metabolism markers in 7453 Korean adults using data from KNHANES, a nationally representative study of the Korean population. In women, consuming two or more cups of coffee per day was inversely associated with elevated HOMA-IR and fasting insulin levels compared to no consumption. By coffee type, women who consumed more black coffee had lower odds of elevated HOMA-IR and fasting insulin levels, showing a significant linear trend. In men or those consuming coffee with sugar and/or cream, no significant associations with glucose metabolism markers were observed.

Consistent with findings from cross-sectional studies [14,16,17,19,20,21,25,36], we observed inverse associations between coffee consumption and elevated HOMA-IR and fasting insulin levels, particularly in women. Both HOMA-IR and fasting insulin levels are closely related to insulin resistance [40]. A meta-analysis of four RCTs found an inverse association between coffee intake and HOMA-IR; however, this association was no longer significant after excluding data from young and healthy individuals [22]. Additionally, we observed no significant associations with other glucose metabolism markers, including elevated fasting plasma glucose, HbA1c, and low HOMA-β, which aligns with previous findings [17,19,21,36]. A study conducted in Sweden that assessed insulin sensitivity using a hyperinsulinemic-euglycemic clamp suggested that the proposed antidiabetic effect of coffee is more likely due to improved insulin sensitivity rather than enhanced beta cell function [21]. Properly functioning beta cells may secrete adequate insulin in response to elevated insulin resistance, helping to maintain normal glucose homeostasis. Moreover, the null association with HbA1c levels may partly result from the low inter-individual variability in HbA1c levels among healthy individuals, which may hinder the detection of a potential relationship [20]. Additionally, a cohort study involving Korean adults aged 40–69 years at baseline reported that consuming ≥2 cups/day of coffee was associated with 20% lower odds of having prediabetes and T2DM combined compared to consuming almost none, whereas consuming <2 cups/day of coffee did not confer this effect [28]. This finding aligns with the intake level associated with insulin resistance in our study. Several cross-sectional studies have suggested that consuming ≥ 3 cups/day of coffee is inversely associated with HOMA-IR [14,16] or impaired glucose tolerance [36], although a large portion of studies treated coffee consumption as a continuous variable in their analyses.

On the other hand, several previous studies reported no significant associations between coffee consumption and elevated HOMA-IR or fasting insulin levels [15,23,24]. The non-significant associations between coffee intake and glucose metabolism markers in two RCTs [23,24] might be attributed to small sample sizes (<130 participants), differences in physiological responses between long-term and short-term coffee intake, potential compliance issues with the coffee intervention or abstinence, or the use of non-dairy creamer with coffee. Furthermore, a Brazilian cross-sectional study that reported non-significant associations found that approximately 60% of participants consumed coffee with added sugar [15].

The exclusive inverse association between black coffee consumption and insulin resistance may be explained by the possibility that adding sugar or cream offsets the antidiabetic effects of bioactive components in coffee, such as caffeine and chlorogenic acids. A review reported that dietary fructose intake strongly contributes to hepatic insulin resistance, with some of its mechanisms being independent of caloric intake and weight gain [41]. A U.S. cohort study indicated that regular sugar-sweetened beverage consumption is positively associated with the progression of prediabetes and insulin resistance [42]. Moreover, a recent cross-sectional study involving Korean adults reported an inverse association between moderate black coffee consumption and metabolic syndrome, whereas coffee with sugar and/or cream showed no significant association [43].

In our study, only women who consumed ≥2 cups/day of coffee had lower odds of elevated HOMA-IR and fasting insulin levels, consistent with findings from several previous studies [16,17]. The underlying mechanism of the sex-specific effect of coffee on insulin sensitivity remains unclear. Low sex-hormone-binding globulin (SHBG) levels, which are increased by estrogens and reduced by androgens, are a strong predictor of T2DM. Previous studies suggested that SHBG may contribute to the inverse association between coffee consumption and T2DM risk in women [44,45]. A review further reported that the inverse association between SHBG and insulin resistance was stronger in women than in men [46]. Additionally, women generally exhibit less variation in lifestyle factors than men, such as lower rates of smoking and alcohol consumption, which may make diet a more influential factor in their glucometabolic status.

The protective effects of coffee in lowering insulin resistance may be further supported by the following possible mechanisms. First, caffeine and chlorogenic acid have demonstrated anti-inflammatory and antioxidant properties in vivo and in vitro [11,12]. Caffeine blocks adenosine receptors and may thereby influence the regulation of reactive oxygen species production. Chlorogenic acids may exert anti-inflammatory effects by inhibiting NF-κB and activating nuclear factor erythroid 2-related factor 2 (Nrf2) [47]. A recent large-scale cohort study involving participants from the U.K. and the Netherlands indicated that lower subclinical inflammation may partially mediate the inverse association between coffee intake and T2DM risk [48]. An animal study demonstrated that chlorogenic acid promotes glucose transport in skeletal muscle via AMPK activation [49]. Moreover, chlorogenic acid may lower glucose absorption by inhibiting sodium-dependent glucose transporters and may reduce hepatic glucose output by inhibiting glucose-6-phosphatase activity [36].

On the other hand, a meta-analysis of seven RCTs suggested that, in the short term, acute caffeine ingestion reduces insulin sensitivity in healthy individuals [26], probably due to increased plasma epinephrine levels. A systematic review of eight clinical trials indicated that caffeinated coffee intake may contribute to unfavorable acute glucose responses; however, an improvement in glucose metabolism was observed in long-term follow-up [50]. Several animal studies reported that long-term caffeine intake is inversely associated with insulin resistance through various mechanisms, including improved insulin/IGF-1 signaling via the induction of insulin receptor substrate 2 or a decrease in circulating catecholamines [51,52].

To the best of our knowledge, this is the first study using KNHANES to examine the associations between coffee consumption and glucose metabolism markers according to coffee type. As this study utilized nationally representative data from the Korean population, our findings can be generalized to Koreans. Our study also has some limitations. Since this study was cross-sectional, causal inferences are limited. Nevertheless, excluding participants with diseases or those following diet therapy for diseases may reduce the likelihood of reverse causation. Furthermore, insulin resistance or hyperinsulinemia is likely to be asymptomatic [17]. Although we adjusted for various potential confounders, including diet quality using the DQI-K and health-related behaviors such as physical activity, smoking status, alcohol consumption, and sleep duration, residual confounding may remain. For example, we could not account for the consumption of other specific foods that may influence insulin resistance, such as bitter melon [53], due to their very low intake frequency in this study. We assessed insulin resistance based on fasting insulin levels and HOMA-IR, which are surrogate measures for insulin resistance. Nevertheless, previous studies have shown that HOMA-IR closely correlates with insulin resistance assessed by the hyperinsulinemic-euglycemic clamp, the gold standard for measuring insulin resistance, and can be reliably used in large-scale epidemiological studies [4,5]. Additionally, we could not account for other coffee types (e.g., decaffeinated coffee) or the amount of caffeine from coffee or other sources, such as tea or energy drinks, due to the limited sample size or data availability. Findings on the association between decaffeinated coffee intake and insulin sensitivity remain inconsistent [17,24,25]. A dose–response meta-analysis of 30 prospective studies suggested that the inverse associations between coffee intake and T2DM risk were comparable for caffeinated and decaffeinated coffee [2]. Furthermore, a study reported that single-nucleotide polymorphisms in genes influencing caffeine metabolism, such as *CYP1A2*, did not modify the inverse association between coffee consumption and cardiometabolic risk [54]. In addition, different devices were used to estimate glycemic parameters, including fasting glucose, fasting insulin, and HbA1c levels, which could have slightly affected our findings. However, measurements conducted by trained staff in a certified laboratory following a standardized protocol likely attenuated any such impact. Additionally, previous research that assessed diet multiple times during follow-up found relatively stable coffee intake patterns and suggested that a single assessment of coffee intake may reasonably reflect medium- to long-term coffee consumption [55].

## 5. Conclusions

In conclusion, consuming two or more cups of black coffee per day is inversely associated with insulin resistance in Korean women. These findings may help elucidate the physiological mechanisms underlying the inverse association between coffee consumption and T2DM risk. Further large-scale prospective studies are needed to clarify the association between various types of coffee consumption and insulin resistance.

## Figures and Tables

**Table 1:** Characteristics of the study population according to categories of total coffee consumption in Korean adults aged 19–64 years ^a^.

Total Coffee Consumption											
	Total		Non-Drinkers		≤1 cup/day		2 cups/day		≥3 cups/day		
	Mean or *n*	SE or %	Mean or *n*	SE or %	Mean or *n*	SE or %	Mean or *n*	SE or %	Mean or *n*	SE or %	*p* ^†^
*n*	7453		2447		1969		1984		1053		
Age (years)	40.60	0.19	36.32	0.31	45.04	0.34	41.23	0.30	42.12	0.38	<0.001 *
Sex (%)											<0.001 *
Men	3298	52.05	1103	53.44	728	44.00	853	50.62	614	64.89	
Women	4155	47.95	1344	46.56	1241	56.00	1131	49.38	439	35.11	
BMI ^a^ (kg/m^2^)	23.57	21, 26	23.26	21, 26	23.55	21, 26	23.68	23, 26	24.08	22, 26	0.021 *
Total coffee (cups/day)	0.98	0.02	0.00	0.00	0.64	0.01	1.41	0.01	3.07	0.05	
Education level (%)											<0.001 *
Middle school or lower	671	6.51	204	5.66	236	8.81	141	5.21	90	6.99	
High school	2915	40.06	1122	47.80	753	39.28	673	34.35	367	33.36	
College or higher	3865	53.44	1120	46.53	980	51.91	1170	60.44	595	59.65	
Monthly household income (%)											<0.001 *
Lowest	563	6.89	232	9.13	130	5.53	126	5.43	75	6.53	
Lower-middle	1581	20.01	549	21.68	420	19.82	378	17.80	234	20.41	
Upper-middle	2386	32.46	762	31.12	630	33.44	653	33.06	341	32.89	
Highest	2906	40.64	896	38.07	785	41.21	825	43.71	400	40.17	
Marital status (%)											<0.001 *
Married	5376	65.99	1392	48.85	1679	81.36	1518	71.34	787	71.10	
Unmarried	2077	34.01	1055	51.15	290	18.64	466	28.66	266	28.90	
Alcohol consumption (%)											0.002 *
<1 serving/day	5629	73.43	1853	74.05	1535	76.10	1495	73.22	746	67.83	
1–<3 servings/day	1183	17.22	379	16.70	279	14.95	336	18.17	189	20.56	
≥3 servings/day	641	9.35	215	9.26	155	8.96	153	8.61	118	11.61	
Smoking status (%)											<0.001 *
Non-smoker	4449	55.61	1576	61.01	1274	60.45	1163	54.22	436	37.22	
Past smoker	1593	23.19	479	21.06	421	23.44	446	24.56	247	25.30	
Current smoker	1411	21.20	392	17.93	274	16.11	375	21.21	370	37.49	
Sleep duration (%)											0.015 *
<6 h/day	3033	43.26	991	43.18	804	42.71	808	43.57	430	43.85	
6–< 8 h/day	3113	40.41	974	38.15	831	41.63	850	41.07	458	42.55	
≥8 h/day	1307	16.32	482	18.67	334	15.66	326	15.36	165	13.61	
Physical activity ^b^ (%)											0.012 *
Low	3925	51.02	1220	48.28	1037	51.22	1077	52.55	591	54.41	
High	3528	48.98	1227	51.72	932	48.78	907	47.45	462	45.59	
Arterial hypertension diagnosis (%)											
No	6716	91.76	2229	93.05	1733	89.72	1799	91.57	955	92.50	0.002 *
Yes	737	8.24	218	6.95	236	10.28	185	8.43	98	7.50	
Family history of diabetes mellitus (%)											0.178
No	5527	75.09	1859	76.82	1451	74.53	1429	73.59	788	74.66	
Yes	1863	24.91	567	23.18	504	25.47	537	26.41	255	25.34	
Supplement intake (%)											<0.001 *
No	3740	51.60	1341	57.23	872	45.11	958	48.63	569	54.52	
Yes	3713	48.40	1106	42.77	1097	54.89	1026	51.37	484	45.48	
Diet quality ^c^ (%)											0.001 *
Poor	3535	44.77	1310	56.93	945	50.71	1068	55.37	595	58.55	
Good	3918	55.23	1137	43.07	1024	49.29	916	44.63	458	41.45	
Energy intake (kcal/day)	1984	12.20	1951	22.22	1911	20.42	1969	20.78	2211	27.88	<0.001 *

^a^ Values are presented as number and weighted percentage for categorical variables, and as mean and standard error for continuous variables. BMI is presented as median and interquartile range, as the data were not normally distributed. ^b^ High physical activity is defined as at least 150 min of moderate activity per week, at least 75 min of vigorous activity per week, or at least 150 min of a combination of moderate and vigorous activity per week, with 1 min of vigorous activity considered equivalent to 2 min of moderate activity. ^c^ Diet quality was assessed using a modified diet quality index for Koreans (DQI-K) and categorized as good (scores of 0–4) or poor (scores of 5–9). ^†^ *p*-values were obtained using the χ^2^ test for categorical variables and analysis of variance (F-test) for continuous variables. The asterisk (*) indicates statistical significance at *p* < 0.05.

**Table 2:** Multivariable-adjusted ORs for glucose metabolism markers according to coffee consumption and type in Korean adults aged 19–64 years.

	Non-Drinkers		≤1 cup/day		2 cups/day		≥3 cups/day		
	OR	95% CI	OR	95% CI	OR	95% CI	OR	95% CI	*p*-Trend
Total coffee									
No. of subjects	2447		1969		1984		1053		
Hyperglycemia									
No. of cases	611		593		576		328		
Model 1 ^a^	1	Ref.	1.00	0.85–1.17	1.12	0.95–1.32	1.05	0.85–1.29	0.402
Model 2 ^b^	1	Ref.	0.94	0.79–1.13	1.08	0.91–1.29	0.99	0.79–1.24	0.727
High fasting insulin									
No. of cases	657		459		465		266		
Model 1	1	Ref.	0.99	0.84–1.17	0.96	0.81–1.14	0.98	0.81–1.19	0.707
Model 2	1	Ref.	0.93	0.76–1.13	0.88	0.73–1.06	0.89	0.70–1.14	0.253
High HOMA-IR									
No. of cases	670		487		482		282		
Model 1	1	Ref.	0.95	0.81–1.12	0.94	0.80–1.11	0.98	0.81–1.18	0.711
Model 2	1	Ref.	0.89	0.73–1.08	0.87	0.73–1.05	0.91	0.72–1.15	0.338
Low HOMA-β									
No. of cases	539		568		484		268		
Model 1	1	Ref.	1.05	0.89–1.24	1.04	0.88–1.23	1.06	0.87–1.29	0.576
Model 2	1	Ref.	1.04	0.88–1.24	1.04	0.86–1.25	1.02	0.82–1.27	0.831
High HbA1c									
No. of cases	689		766		674		372		
Model 1	1	Ref.	1.09	0.93–1.27	1.13	0.97–1.32	1.12	0.93–1.36	0.160
Model 2	1	Ref.	1.07	0.90–1.27	1.13	0.96–1.34	1.10	0.90–1.35	0.231
Black coffee									
No. of subjects	2447		1005		867		283		
Hyperglycemia									
No. of cases	611		278		220		67		
Model 1	1	Ref.	0.97	0.80–1.19	1.08	0.88–1.33	0.84	0.60–1.19	0.682
Model 2	1	Ref.	0.91	0.73–1.12	0.98	0.78–1.24	0.77	0.53–1.12	0.257
High fasting insulin									
No. of cases	657		216		212		71		
Model 1	1	Ref.	0.85	0.70–1.04	0.93	0.75–1.14	0.88	0.64–1.21	0.305
Model 2	1	Ref.	0.79 *	0.62–1.00	0.82	0.64–1.04	0.81	0.56–1.17	0.086
High HOMA-IR									
No. of cases	670		226		214		72		
Model 1	1	Ref.	0.81 *	0.66–1.00	0.92	0.75–1.14	0.89	0.65–1.21	0.300
Model 2	1	Ref.	0.74 *	0.58–0.95	0.81	0.64–1.03	0.83	0.58–1.19	0.092
Low HOMA-β									
No. of cases	539		290		196		52		
Model 1	1	Ref.	1.14	0.93–1.39	1.05	0.83–1.31	0.82	0.56–1.21	0.573
Model 2	1	Ref.	1.16	0.94–1.43	1.08	0.85–1.36	0.79	0.52–1.21	0.557
High HbA1c									
No. of cases	689		355		247		84		
Model 1	1	Ref.	1.03	0.84–1.26	1.06	0.86–1.31	1.26	0.91–1.76	0.174
Model 2	1	Ref.	1.01	0.82–1.25	1.05	0.83–1.32	1.28	0.89–1.83	0.217
Coffee with sugar and/or cream									
No. of subjects	2447		832		712		467		
Hyperglycemia									
No. of cases	611		282		231		182		
Model 1	1	Ref.	1.05	0.85–1.30	1.06	0.86–1.32	1.22	0.92–1.61	0.175
Model 2	1	Ref.	0.95	0.76–1.20	1.01	0.81–1.27	1.08	0.80–1.46	0.642
High fasting insulin									
No. of cases	657		216		170		116		
Model 1	1	Ref.	1.23	0.98–1.54	1.05	0.83–1.34	1.07	0.81–1.40	0.609
Model 2	1	Ref.	1.11	0.86–1.44	1.00	0.76–1.30	0.96	0.68–1.35	0.825
High HOMA-IR									
No. of cases	670		234		178		134		
Model 1	1	Ref.	1.18	0.95–1.46	1.00	0.79–1.25	1.08	0.83–1.41	0.676
Model 2	1	Ref.	1.08	0.84–1.40	0.95	0.74–1.24	1.01	0.73–1.40	0.935
Low HOMA-β									
No. of cases	539		243		180		148		
Model 1	1	Ref.	0.91	0.73–1.12	0.94	0.75–1.18	1.18	0.92–1.51	0.361
Model 2	1	Ref.	0.88	0.70–1.10	0.90	0.70–1.15	1.15	0.87–1.51	0.635
High HbA1c									
No. of cases	689		357		279		197		
Model 1	1	Ref.	1.15	0.94–1.40	1.22	1.00–1.49	1.16	0.89–1.51	0.111
Model 2	1	Ref.	1.10	0.89–1.37	1.21	0.98–1.48	1.10	0.83–1.46	0.242

HOMA-IR, homeostatic model assessment of insulin resistance; HOMA-β, homeostatic model assessment of beta-cell function; Ref., reference. ^a^ Model 1 was adjusted for age and sex. ^b^ Model 2 was adjusted for age, sex, BMI, education level, monthly household income, marital status, alcohol consumption, smoking status, sleep duration, physical activity, arterial hypertension diagnosis, family history of diabetes mellitus, supplement intake, a modified diet quality index for Koreans, and total daily energy intake. * The asterisk indicates statistical significance at *p* < 0.05.

**Table 3:** Multivariable-adjusted ORs for high fasting insulin and HOMA-IR according to coffee consumption and type in Korean adults aged 19–64 years by sex.

	Non-Drinkers		≤1 cup/day		2 cups/day		≥3 cups/day		
	OR	95% CI	OR	95% CI	OR	95% CI	OR	95% CI	*p*-Trend
Women									
Total coffee									
No. of subjects	1344		1241		1131		439		
High fasting insulin									
No. of cases	329		250		216		90		
Model 1 ^a^	1	Ref.	0.99	0.79–1.24	0.78 *	0.63–0.97	0.86	0.63–1.18	0.083
Model 2 ^b^	1	Ref.	0.92	0.71–1.21	0.70 *	0.54–0.91	0.64 *	0.43–0.93	0.003 *
High HOMA-IR									
No. of cases	320		266		216		86		
Model 1	1	Ref.	1.00	0.81–1.25	0.80 *	0.64–1.00	0.87	0.63–1.21	0.121
Model 2	1	Ref.	0.95	0.74–1.23	0.73 *	0.56–0.96	0.66 *	0.44–0.99	0.009 *
Black coffee									
No. of subjects	1344		660		479		136		
High fasting insulin									
No. of cases	329		121		91		24		
Model 1	1	Ref.	0.84	0.64–1.10	0.74	0.55–1.00	0.62	0.36–1.06	0.017 *
Model 2	1	Ref.	0.79	0.57–1.09	0.63 *	0.44–0.90	0.58	0.31–1.09	0.009 *
High HOMA-IR									
No. of cases	320		127		88		21		
Model 1	1	Ref.	0.85	0.66–1.11	0.74	0.55–1.01	0.61	0.34–1.08	0.018 *
Model 2	1	Ref.	0.81	0.60–1.10	0.64 *	0.45–0.93	0.59	0.30–1.16	0.016 *
Coffee with sugar and/or cream									
No. of subjects	1344		475		374		132		
High fasting insulin									
No. of cases	329		110		80		31		
Model 1	1	Ref.	1.31	0.96–1.80	0.95	0.68–1.32	1.19	0.72–1.96	0.657
Model 2	1	Ref.	1.13	0.79–1.61	0.80	0.55–1.16	0.69	0.39–1.21	0.149
High HOMA-IR									
No. of cases	320		120		81		33		
Model 1	1	Ref.	1.35 *	1.00–1.81	1.00	0.72–1.38	1.31	0.80–2.13	0.363
Model 2	1	Ref.	1.17	0.83–1.67	0.89	0.62–1.27	0.83	0.48–1.46	0.505
Men									
Total coffee									
No. of subjects	1103		728		853		614		
High fasting insulin									
No. of cases	328		209		249		176		
Model 1	1	Ref.	0.98	0.77–1.26	1.12	0.88–1.42	1.05	0.82–1.35	0.498
Model 2	1	Ref.	0.94	0.71–1.25	1.04	0.79–1.37	1.03	0.75–1.41	0.725
High HOMA-IR									
No. of cases	350		221		266		196		
Model 1	1	Ref.	0.89	0.70–1.14	1.06	0.84–1.33	1.02	0.80–1.31	0.599
Model 2	1	Ref.	0.85	0.64–1.13	0.99	0.77–1.28	1.02	0.76–1.38	0.707
Black coffee									
No. of subjects	1103		345		388		147		
High fasting insulin									
No. of cases	328		95		121		47		
Model 1	1	Ref.	0.85	0.63–1.15	1.08	0.82–1.43	1.04	0.70–1.56	0.678
Model 2	1	Ref.	0.82	0.58–1.16	0.99	0.71–1.39	0.98	0.61–1.56	0.580
High HOMA-IR									
No. of cases	350		99		126		51		
Model 1	1	Ref.	0.76	0.55–1.04	1.06	0.80–1.39	1.05	0.71–1.55	0.717
Model 2	1	Ref.	0.72	0.49–1.04	0.95	0.69–1.31	0.98	0.62–1.53	0.872
Coffee with sugar and/or cream									
No. of subjects	1103		357		338		335		
High fasting insulin									
No. of cases	328		106		90		85		
Model 1	1	Ref.	1.16	0.83–1.61	1.14	0.81–1.59	1.03	0.74–1.42	0.776
Model 2	1	Ref.	1.10	0.76–1.60	1.15	0.78–1.69	1.00	0.66–1.51	0.886
High HOMA-IR									
No. of cases	350		114		97		101		
Model 1	1	Ref.	1.04	0.76–1.43	0.98	0.71–1.35	0.99	0.73–1.35	0.916
Model 2	1	Ref.	1.00	0.70–1.43	0.98	0.67–1.44	0.99	0.68–1.45	0.953

HOMA-IR, homeostatic model assessment of insulin resistance; HOMA-β, homeostatic model assessment of beta-cell function; Ref., reference. ^a^ Model 1 was adjusted for age. ^b^ Model 2 was adjusted for age, BMI, education level, monthly household income, marital status, alcohol consumption, smoking status, sleep duration, physical activity, arterial hypertension diagnosis, family history of diabetes mellitus, supplement intake, a modified diet quality index for Koreans, and total daily energy intake. * The asterisk indicates statistical significance at *p* < 0.05.

## Data Availability

The relevant data are publicly available on the KNHANES website: https://knhanes.kdca.go.kr/knhanes/eng/main.do (accessed on 7 November 2024).

## Supplementary Materials

The following supporting information can be downloaded at https://www.mdpi.com/article/10.3390/nu17091484/s1. Table S1: Multivariable-adjusted ORs for high fasting insulin and HOMA-IR according to coffee consumption and type in Korean women aged 19–64 years, based on five sequential adjustment models. Table S2: Multivariable-adjusted ORs for high HOMA-IR according to coffee consumption and type in Korean adults aged 19–64 years, with the study population restricted to individuals with fasting glucose levels < 6.99 mmol/L (<126 mg/dL). Table S3: Multivariable-adjusted ORs for glucose metabolism markers according to coffee consumption in Korean adults aged 65 years and older.

## Abbreviations

The following abbreviations are used in this manuscript:

DQI-K	A modified diet quality index for Koreans
HOMA-β	Homeostatic model assessment of beta-cell function
HOMA-IR	Homeostasis model assessment of insulin resistance
KNHANES	Korea National Health and Nutrition Examination Survey
