# Supplementary material for: Association Between Coffee Consumption and Glucose Metabolism Markers in Korean Adults
Source: Nutrients. 2025 Apr 28;17(9):1484. doi: 10.3390/nu17091484 (PMC12073867; doi:10.3390/nu17091484)
Supplement: Supplementary file 1 [file nutrients-17-01484-s001.zip › nutrients-3598949-supplementary.pdf]

Table S1. Multivariable-adjusted ORs for high fasting insulin and HOMA-IR according to coffee consumption and type in Korean women aged 19–64 years, based on five sequential adjustment models

	Non-drinkers		≤1 cup/day		2 cups/day		≥3 cups/day	
	OR	95% CI	OR	95% CI	OR	95% CI	OR	95% CI
Women								
Total coffee								
No. of subjects	1344		1241		1131		439	
High fasting insulin								
No. of cases	329		250		216		90	
Model 1[a]	1	Ref.	0.99	0.79–1.24	0.78*	0.63–0.97	0.86	0.63–1.18
Model 2[b]	1	Ref.	1.03	0.82–1.30	0.84	0.67–1.05	0.88	0.64–1.21
Model 3[c]	1	Ref.	0.91	0.70–1.18	0.70*	0.54–0.91	0.62*	0.42–0.91
Model 4[d]	1	Ref.	0.92	0.70–1.20	0.70*	0.54–0.90	0.63*	0.43–0.93
Model 5[e]	1	Ref.	0.92	0.71–1.21	0.70*	0.54–0.91	0.64*	0.43–0.93
High HOMA-IR								
No. of cases	320		266		216		86	
Model 1	1	Ref.	1.00	0.81–1.25	0.80	0.64–1.00	0.87	0.63–1.21
Model 2	1	Ref.	1.06	0.84–1.32	0.86	0.69–1.09	0.90	0.65–1.25
Model 3	1	Ref.	0.93	0.72–1.20	0.73*	0.56–0.95	0.63*	0.43–0.94
Model 4	1	Ref.	0.94	0.73–1.22	0.73*	0.56–0.95	0.66*	0.44–0.98
Model 5	1	Ref.	0.95	0.74–1.23	0.73*	0.56–0.96	0.66*	0.44–0.99
Black coffee								
No. of subjects	1344		660		479		136	
High fasting insulin								
No. of cases	329		121		91		24	
Model 1	1	Ref.	0.84	0.64–1.10	0.74	0.55–1.00	0.62	0.36–1.06
Model 2	1	Ref.	0.89	0.68–1.16	0.80	0.59–1.10	0.68	0.39–1.16
Model 3	1	Ref.	0.77	0.56–1.06	0.64*	0.45–0.91	0.57	0.30–1.08
Model 4	1	Ref.	0.78	0.57–1.08	0.62*	0.43–0.89	0.58	0.31–1.10
Model 5	1	Ref.	0.79	0.57–1.09	0.63*	0.44–0.90	0.58	0.31–1.09
High HOMA-IR								
No. of cases	320		127		88		21	
Model 1	1	Ref.	0.85	0.66–1.11	0.74	0.55–1.01	0.61	0.34–1.08
Model 2	1	Ref.	0.91	0.70–1.18	0.82	0.60–1.12	0.67	0.38–1.19
Model 3	1	Ref.	0.79	0.58–1.07	0.66*	0.46–0.95	0.58	0.29–1.14
Model 4	1	Ref.	0.80	0.59–1.10	0.64*	0.44–0.92	0.59	0.30–1.17
Model 5	1	Ref.	0.81	0.60–1.10	0.64*	0.45–0.93	0.59	0.30–1.16
Coffee with sugar and/or cream								
No. of subjects	1344		475		374		132	
High fasting insulin								
No. of cases	329		110		80		31	
Model 1	1	Ref.	1.31	0.96–1.80	0.95	0.68–1.32	1.19	0.72–1.96
Model 2	1	Ref.	1.29	0.94–1.76	0.91	0.65–1.28	1.03	0.62–1.70
Model 3	1	Ref.	1.13	0.80–1.59	0.80	0.55–1.14	0.68	0.39–1.18
Model 4	1	Ref.	1.12	0.79–1.60	0.81	0.56–1.17	0.70	0.40–1.23
Model 5	1	Ref.	1.13	0.79–1.61	0.80	0.55–1.16	0.69	0.39–1.21
High HOMA-IR								

No. of cases	320		120		81		33	
Model 1	1	Ref.	1.35	1.00–1.81	1.00	0.72–1.38	1.31	0.80–2.13
Model 2	1	Ref.	1.32	0.98–1.78	0.98	0.70–1.37	1.16	0.70–1.91
Model 3	1	Ref.	1.17	0.83–1.64	0.87	0.61–1.24	0.79	0.46–1.36
Model 4	1	Ref.	1.17	0.82–1.66	0.89	0.63–1.27	0.83	0.47–1.45
Model 5	1	Ref.	1.17	0.83–1.67	0.89	0.62–1.27	0.83	0.48–1.46

HOMA-IR, homeostatic model assessment of insulin resistance; HOMA-β, homeostatic model assessment of beta-cell function; Ref., reference.

[a] Model 1 was adjusted for age.

[b] Model 2 was adjusted for age, education level, monthly household income, marital status, alcohol consumption, smoking status, sleep duration, physical activity, and total daily energy intake.

[c] Model 3 was adjusted for age, education level, monthly household income, marital status, alcohol consumption, smoking status, sleep duration, physical activity, total daily energy intake, and BMI.

[d] Model 4 was adjusted for age, education level, monthly household income, marital status, alcohol consumption, smoking status, sleep duration, physical activity, total daily energy intake, BMI, hypertension diagnosis, and family history of diabetes.

[e] Model 5 was adjusted for age, education level, monthly household income, marital status, alcohol consumption, smoking status, sleep duration, physical activity, total daily energy intake, BMI, hypertension diagnosis, family history of diabetes, supplement intake, and a modified diet quality index for Koreans.

[*] The asterisk indicates statistical significance at $p < 0.05$.

Table S2. Multivariable-adjusted ORs for high HOMA-IR according to coffee consumption and type in Korean adults aged 19–64 years, with the study population restricted to individuals with fasting glucose levels <6.99 mmol/L (<126 mg/dL)

	Non-drinkers		≤1 cup/day		2 cups/day		≥3 cups/day	
	OR	95% CI	OR	95% CI	OR	95% CI	OR	95% CI
Adults								
Total coffee								
No. of subjects	2383		1922		1921		1021	
No. of cases	613		447		437		256	
Model 1[a]	1	Ref.	0.99	0.83–1.18	0.98	0.82–1.16	0.98	0.81–1.20
Model 2[b]	1	Ref.	0.90	0.73–1.11	0.89	0.73–1.07	0.91	0.71–1.16
Black coffee								
No. of subjects	2383		985		848		280	
No. of cases	613		211		198		69	
Model 1	1	Ref.	0.86	0.70–1.06	0.94	0.76–1.17	0.92	0.67–1.26
Model 2	1	Ref.	0.77*	0.60–0.99	0.82	0.63–1.05	0.83	0.57–1.21
Coffee with sugar and/or cream								
No. of subjects	2383		807		683		444	
No. of cases	613		211		158		115	
Model 1	1	Ref.	1.21	0.96–1.52	1.03	0.81–1.31	1.07	0.81–1.41
Model 2	1	Ref.	1.08	0.83–1.42	0.99	0.75–1.29	1.01	0.72–1.42
Women								
Total coffee								
No. of subjects	1321		1221		1109		432	
No. of cases	301		248		198		81	
Model 1	1	Ref.	1.02	0.81–1.28	0.80	0.64–1.01	0.87	0.62–1.21
Model 2	1	Ref.	0.92	0.71–1.21	0.72*	0.55–0.95	0.65*	0.43–0.97
Black coffee								
No. of subjects	1321		652		472		136	
No. of cases	301		121		82		21	
Model 1	1	Ref.	0.89	0.68–1.15	0.74	0.54–1.02	0.64	0.36–1.14
Model 2	1	Ref.	0.81	0.59–1.11	0.63*	0.43–0.92	0.60	0.30–1.18
Coffee with sugar and/or cream								
No. of subjects	1321		465		365		126	
No. of cases	301		110		73		28	
Model 1	1	Ref.	1.35	0.99–1.86	1.00	0.71–1.41	1.15	0.69–1.94
Model 2	1	Ref.	1.11	0.76–1.61	0.88	0.61–1.28	0.75	0.41–1.38
Men								
Total coffee								
No. of subjects	1062		701		812		589	
No. of cases	312		199		239		175	

Model 1	1	Ref.	0.94	0.73–1.22	1.12	0.88–1.43	1.05	0.81–1.37
Model 2	1	Ref.	0.89	0.66–1.21	1.04	0.79–1.36	1.05	0.77–1.44
Black coffee								
No. of subjects	1062		333		376		144	
No. of cases	312		90		116		48	
Model 1	1	Ref.	0.82	0.59–1.14	1.10	0.82–1.47	1.08	0.72–1.62
Model 2	1	Ref.	0.76	0.52–1.13	0.98	0.70–1.37	0.99	0.62–1.59
Coffee with sugar and/or cream								
No. of subjects	1062		342		318		318	
No. of cases	312		101		85		87	
Model 1	1	Ref.	1.09	0.78–1.53	1.05	0.75–1.46	1.02	0.74–1.42
Model 2	1	Ref.	1.06	0.73–1.54	1.06	0.71–1.58	1.04	0.70–1.55

HOMA-IR, homeostatic model assessment of insulin resistance; HOMA-β, homeostatic model assessment of beta-cell function; Ref., reference.

[a] Model 1 was adjusted for age and sex.

[b] Model 2 was adjusted for age, sex, BMI, education level, monthly household income, marital status, alcohol consumption, smoking status, sleep duration, physical activity, hypertension diagnosis, family history of diabetes, supplement intake, a modified diet quality index for Koreans, and total daily energy intake.

[*] The asterisk indicates statistical significance at $p < 0.05$.

Table S3. Multivariable-adjusted ORs for glucose metabolism markers according to coffee consumption in Korean adults aged 65 years and older

	Non-drinkers		≤1 cup/day		2 cups/day		≥3 cups/day		
	OR	95% CI	OR	95% CI	OR	95% CI	OR	95% CI	*p*-trend
Total coffee									
No. of subjects	568		581		286		99		
Hyperglycemia									
No. of cases	261		281		140		47		
Model 1[a]	1	Ref.	1.17	0.90–1.50	1.06	0.77–1.46	1.07	0.64–1.79	0.686
Model 2[b]	1	Ref.	1.25	0.93–1.68	0.99	0.69–1.42	1.04	0.61–1.76	0.921
High fasting insulin									
No. of cases	151		146		63		17		
Model 1	1	Ref.	0.95	0.69–1.30	0.85	0.58–1.25	0.66	0.37–1.18	0.147
Model 2	1	Ref.	0.81	0.58–1.15	0.61*	0.39–0.95	0.56*	0.31–0.99	0.009*
High HOMA-IR									
No. of cases	150		155		65		19		
Model 1	1	Ref.	1.02	0.75–1.40	0.82	0.56–1.19	0.75	0.42–1.32	0.190
Model 2	1	Ref.	0.96	0.68–1.35	0.60*	0.38–0.93	0.69	0.39–1.21	0.023*
Low HOMA-β									
No. of cases	151		136		69		28		
Model 1	1	Ref.	0.80	0.59–1.09	0.74	0.51–1.08	1.19	0.64–2.21	0.701
Model 2	1	Ref.	0.94	0.68–1.29	0.96	0.64–1.44	1.34	0.66–2.74	0.646
High HbA1c									
No. of cases	350		380		167		69		
Model 1	1	Ref.	1.23	0.90–1.70	0.87	0.62–1.24	1.38	0.74–2.57	0.720
Model 2	1	Ref.	1.23	0.88–1.70	0.82	0.57–1.18	1.37	0.69–2.72	0.887

HOMA-IR, homeostatic model assessment of insulin resistance; HOMA-β, homeostatic model assessment of beta-cell function; Ref., reference.

[a] Model 1 was adjusted for age and sex.

[b] Model 2 was adjusted for age, sex, BMI, education level, monthly household income, marital status, alcohol consumption, smoking status, sleep duration, physical activity, hypertension diagnosis, family history of diabetes, supplement intake, a modified diet quality index for Koreans, and total daily energy intake.

* The asterisk indicates statistical significance at $p < 0.05$.
